# Comparable assessment of adolescent repeated physical or psychological stress effects on adult cardiac performance in female rats

**DOI:** 10.1038/s41598-023-43721-7

**Published:** 2023-09-29

**Authors:** Monireh-Sadat Mousavi, Sogol Meknatkhah, Alireza Imani, Parham Geramifar, Gholamhossein Riazi

**Affiliations:** 1 Laboratory of Neuro-Organic Chemistry, Institute of Biochemistry and Biophysics (IBB), University of Tehran, Tehran, Iran; 2 Department of Physiology, School of Medicine, Tehran University of Medical Sciences, Tehran, Iran; 3 Research Center for Nuclear Medicine, Shariati Hospital, Tehran University of Medical Sciences, Tehran, Iran

**Keywords:** Biochemistry, Molecular biology, Physiology, Psychology, Diseases, Health care

## Abstract

Extensive evidence highlights a robust connection between various forms of chronic stress and cardiovascular disease (CVD). In today's fast-paced world, with chronic stressors abound, CVD has emerged as a leading global cause of mortality. The intricate interplay of physical and psychological stressors triggers distinct neural networks within the brain, culminating in diverse health challenges. This study aims to discern the unique impacts of chronic physical and psychological stress on the cardiovascular system, unveiling their varying potencies in precipitating CVD. Twenty-one adolescent female rats were methodically assigned to three groups: (1) control (n = 7), (2) physical stress (n = 7), and (3) psychological stress (n = 7). Employing a two-compartment enclosure, stressors were administered to the experimental rats over five consecutive days, each session lasting 10 min. After a 1.5-month recovery period post-stress exposure, a trio of complementary techniques characterized by high specificity or high sensitivity were employed to meticulously evaluate CVD. Echocardiography and single-photon emission computed tomography (SPECT) were harnessed to scrutinize left ventricular architecture and myocardial viability, respectively. Subsequently, the rats were ethically sacrificed to facilitate heart removal, followed by immunohistochemistry staining targeting glial fibrillary acidic protein (GFAP). Rats subjected to psychological stress showed a wider range of significant cardiac issues compared to control rats. This included left ventricular hypertrophy [IVSd: 0.1968 ± 0.0163 vs. 0.1520 ± 0.0076, P < 0.05; LVPWd: 0.2877 ± 0.0333 vs. 0.1689 ± 0.0057, P < 0.01; LVPWs: 0.3180 ± 0.0382 vs. 0.2226 ± 0.0121, P < 0.05; LV-mass: 1.283 ± 0.0836 vs. 1.000 ± 0.0241, P < 0.01], myocardial ischemia [21.30% vs. 32.97%, P < 0.001], and neuroinflammation. This outcome underscores the imperative of prioritizing psychological well-being during adolescence, presenting a compelling avenue to curtail the prevalence of CVD in adulthood. Furthermore, extending such considerations to individuals grappling with CVD might prospectively enhance their overall quality of life.

## Introduction

Cardiovascular disease (CVD), which claims approximately 17.9 million lives worldwide annually, stands as the foremost global cause of mortality^1^. Numerous clinical investigations have illuminated the adverse consequences of diverse stress forms on cardiovascular well-being^2–4^. The effects of stress are contingent upon factors such as the organism's age, sex, nature, intensity, and duration of the stressor, leading to a diverse array of physiological responses.

Age and sex disparities significantly influence stress-triggered physiological alterations; notably, prepubertal^5^ or adolescent^6^ females^7,8^ tend to exhibit heightened susceptibility to stress. This heightened vulnerability in prepubertal and adolescent stages can be attributed to the pivotal biological developments transpiring during this crucial transitional life phase^9,10^.

Beyond age and sex influences, research has unveiled the bidirectional influence of stress on immune function, contingent on its duration and intensity. Acute stress generally augments immune activity, whereas chronic stress tends to suppress immune responses^11^.

Categorically, stress manifests in two distinct forms: physical and psychological^12,13^. Physical stress pertains to the application of force on a specific biological tissue region, prompting bodily reactions such as harm, pain, injury, hypertrophy, atrophy, or even death. Such forces encompass perilous circumstances like electrical shocks, traumatic injuries, strenuous physical labor, electromagnetic fields, environmental pollutants, oxygen deprivation, hypoglycemia, dehydration, infections, and dietary insufficiencies^14^. In contrast, psychological stress involves a unique interplay between an individual and their environment, subjectively perceived as distinct emotions, cognitions, or perceptions, surpassing the person's available coping resources and capacities. This realm encompasses emotional states like fear, worry, sadness, resentment, anger, anxiety, and panic attack^15–17^.

While psychological stress, intangible to the naked eye or detectors, has historically been underestimated relative to physical stress, it has garnered significant recent attention^18,19^. The brain distinguishes between physical and psychological stress through the activation of diverse neural networks, resulting in disparate health outcomes^20^. In this context, our prior research has examined the effects of repeated psychological or physical stress during adolescence on multiple sclerosis pathology^21–23^, cardiac natriuretic peptide receptor 3 (NPR3) as a cardiovascular disease biomarker^24^, cognitive function, oxidative stress, and heart rate^25^ in adult female rats. Nevertheless, a comprehensive understanding of the distinct impacts of chronic physical versus psychological stresses on driving CVD necessitates deeper exploration through integrated techniques with high sensitivity and specificity.

## Objectives

While prior research has delved into the enduring impacts of chronic stress during adolescence on cardiac function, a comprehensive understanding of how distinct stressor types yield long-term consequences remains relatively uncharted. Consequently, our objective was to disentangle the enduring repercussions of physical and psychological stressors on both the structural and functional aspects of the heart. This study's core premise postulates that psychological well-being, often overshadowed by physical health, exerts a more robust influence on cardiovascular health. By juxtaposing the enduring, unique impacts of prolonged physical and psychological stresses on cardiac performance, we aim to discern their varying potencies in precipitating subsequent cardiovascular disease (CVD).

## Methods

In this investigation, we employed a two-compartment box system to induce stress^26,27^. Physical stress was instigated by subjecting rats to electrical footshocks. Psychological stress was induced in rats that observed the physical harm and ensuing distress of their neighboring counterparts within the two-compartment box. To assess distinct cardiac dysfunctions, we utilized non-invasive techniques—namely, "echocardiography" and "myocardial perfusion imaging" via single-photon emission computerized tomography (SPECT). These techniques were pivotal in evaluating left ventricular architecture and myocardial ischemia, respectively^28,29^. Furthermore, we conducted immunohistochemistry (IHC) staining for glial fibrillary acidic protein (GFAP) on intrinsic cardiac ganglia (ICG) situated in the posterior wall of the left atrium^30^. This assessment aimed to gauge ICG neuroinflammation/dysregulation. The study findings offered comprehensive insights into the persistent stress-induced cardiac malfunctions resulting from chronic physical or psychological stress. All methods are reported in accordance with ARRIVE guidelines (https://arriveguidelines.org).

### Animals

A total of twenty-one female Wistar rats, aged 21 days postnatal (PND 21), were procured from the University of Tehran's animal house. They were acclimatized for two weeks under standard environmental conditions, including a 12-day/12-night light cycle and a temperature-controlled room (21–24 °C). The rats had free access to standard food and water throughout this adaptation period. The experimental protocols adhered to the guidelines outlined in the US National Institutes of Health Guide for the Care and Use of Laboratory Animals (NIH Publications No. 85-23, revised 2011). These procedures were further endorsed by the Institutional Ethics Committee (IEC) on Laboratory Animal Care at the University of Tehran (Tehran, Iran, Approval No. 30486). Diligent efforts were made to minimize the number of animals used and their discomfort throughout the course of the experiments.

### Experimental design

**Figure 1:**
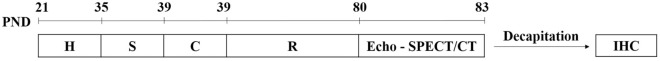
Schematic diagram of the experimental design (three groups, each N = 7). *H* Habituation (PND 21–35), *S* Stress induction (PND 35–39), *C* Corticosterone measurement (PND 39), *R* Rest (PND 39–80), *Echo* 3-D Echocardiography (PND 81), *SPECT/CT* Single photon emission computed tomography/computed tomography (PND 82); Decapitation of experimental rats (PND 83), *IHC* Immunohistochemistry.

Upon reaching PND 21, the rats were randomly categorized into three groups, each comprising 7 rats: control, physical stress, and psychological stress. Following a two-week acclimatization period (PND 21–35), the experimental groups were subjected to physical and psychological stressors for five consecutive days (PND 35–39). On PND 39, serum corticosterone concentration was measured to validate the induction of stress. Subsequently, the rats were returned to their home cages for a 6-week period leading up to adulthood. During PND 81–83, echocardiography and SPECT/CT assessments were conducted to evaluate each group's cardiac performance. On PND 83, the rats were euthanized for immunohistochemical analysis of GFAP (Fig. 1).

### Rat estrous cycle phases synchronization

To synchronize the estrous cycle phases of the female rats, a mature male rat was placed in a small cage within each group's enclosure. Male urinary pheromones are known to influence and harmonize the female rat's estrous cycle^31^. The stages of the estrous cycle were determined through vaginal smear cytology before each test^32^. The female rats were matched for estrous cycle stages, with any disparities managed by scheduling tests slightly earlier or later for those with different stages.

### Induction of physical and psychological stress

A two-compartment box measuring 30 cm × 25 cm × 30 cm, featuring two equally sized chambers separated by a transparent perforated Plexiglas partition, served as the platform for stress induction^26,27^. To elaborate, one chamber's floor was connected to an electric generator (Tajhiz Gostar Omide Iranian CO., Iran) to deliver electrical current (0.25 mA, 50 Hz, 1-s duration, 10 shocks over a 10-min period with randomized intervals) for the physical stress group. The other compartment remained isolated, providing a non-footshock environment for rats in the psychological stress group. The perforated partition enabled rats in the psychological stress group to witness the behavioral and emotional cues (visual, auditory, and olfactory sensations) emitted by the physically stressed rats. Before each stress session for a psychological/physical stress rat pair, a 3-min adaptation period was allotted. Control rats were also placed in the two-compartment box in pairs for the same 10-min period without exposure to footshocks. Following each pair trial, the box was thoroughly cleaned with 70% ethanol.

### Serum corticosterone measurement

Following the final stress exposure session, serum corticosterone levels, serving as an indicator of the stress response, were measured in duplicate aliquots using a commercial ELISA kit (DRG International, USA; Cat. No. EIA-5186) according to the manufacturer's instructions. The assay's detection limit was 4.1 ng/ml, and intra-assay and inter-assay coefficient of variation means were 5.3% and 8.2%, respectively.

### Echocardiography

During PND 81, echocardiograms were conducted using a GE Voluson 730 Pro device from the Kretztechnik Company, Austria. The rats were anesthetized with ketamine–xylazine anesthesia [80–8 mg/kg, intraperitoneal] and their chest area was shaved. Data averages were derived from three consecutive cardiac cycles. M-mode tracings facilitated the measurement of echocardiographic parameters, including interventricular septum thicknesses in diastole (IVSd), left ventricular diameter in diastole (LVDd), left ventricular posterior wall thickness in diastole (LVPWd), interventricular septum thicknesses in systole (IVSs), left ventricular diameter in systole (LVDs), left ventricular posterior wall thickness in systole (LVPWs), left ventricular ejection fraction (LVEF), fractional shortening (FS), diastolic volume (DV), systolic volume (SV), stroke volume, cardiac output (CO), and left ventricular mass (LVM). The first eight parameters (IVSd, LVDd, LVPWd, IVSs, LVDs, LVPWs, LVEF, and FS) were extracted from M-mode images using MATLAB R2011b software. The remaining data (DV, SV, stroke volume, CO, and LVM) were computed using formulas as follows: DV = 1.047 × (LVDd)^3^, SV = 1.047 × (LVDd)^3^, stroke volume = DV − SV, CO = stroke volume × heart rate, and LVM = 1.04 × [(LVDd + LVPW + IVS)^3^ − (LVDd)^3^] × 0.8 + 0.6.

### SPECT/CT imaging

On PND 82, SPECT/CT scans were performed using a SIEMENS Symbia T clinical scanner. Three rats from each group were randomly selected by an observer blinded to the study. Following ketamine–xylazine anesthesia [80–8 mg/kg, intraperitoneal], 1.5 mCi of ^99^mTc-sestamibi radiotracer (Technetium-99 2-methoxy-isobutyl-isonitrile)^33^ was injected through the rats' lateral tail veins. Additional anesthesia was administered during the procedure as required.

Myocardial perfusion SPECT/CT imaging using ^99^mTc-sestamibi was conducted 45 to 90 min post-radiotracer injection. Each imaging session accommodated two rats within the imaging field of view, and CT scans were executed for anatomical reference and attenuation correction (spatial resolution 1.25 mm, 80 kV, 150 mAs), with a total CT scan duration of 10 s. SPECT data were acquired with a non-circular orbit. Reconstruction employed the flash 3D algorithm with 10 iterations and 10 subsets. Transmission data were reconstructed into an equally-sized matrix through filtered back projection, yielding co-registered image sets.

### Immunohistochemistry

On PND 83, rats were anesthetized with diethyl ether inhalation, then perfused transcardially with phosphate-buffered saline (PBS) followed by fixation in 4% paraformaldehyde in 0.2 M phosphate buffer (pH 7.4). After decapitating the animals, their hearts were promptly excised and placed in 10% formalin for subsequent immunohistochemical analysis of GFAP, following a protocol detailed in our prior study^34^. Tissue sections measuring 4 μm in thickness, extracted from the posterior wall of the left atrium, were subjected to immunohistochemistry. This involved incubation with a diluted primary antibody specific to GFAP (rabbit polyclonal antibody, DAKO, code IS524), visualization of primary antibodies using the Envision kit (DAKO, Carpinteria, CA), and use of a secondary anti-mouse antibody-coated polymer peroxidase complex. The prepared slides were then examined using the B-383PL OPTIKA light microscope.

### Statistics

Statistical analyses were performed using the GraphPad InStat 3 statistical software (GraphPad InStat Software, San Diego, CA). Data were subjected to one-way ANOVA followed by Tukey's posthoc test for comparisons, and results were presented as mean ± SEM. P-values below 0.05 were deemed statistically significant.

### Transparency and openness

Materials described in the method section, as well as raw and/or processed data underpinning the study's conclusions, are available upon request via email to the corresponding author. This study was not preregistered. In this study, we experimented by using a two-compartment box system for stress induction^26,27^. Physical stress was induced by exposure to electrical footshocks. Psychological stress was induced in rats that witnessed physical harm and the consequent suffering of their neighboring counterparts in the two-compartment box system. To assess different cardiac dysfunctions, “echocardiography” and “myocardial perfusion imaging” using single-photon emission computerized tomography (SPECT) have provided useful non-invasive techniques^28,29^ evaluating left ventricular architecture and myocardial ischemia, respectively. Moreover, immunohistochemistry (IHC) staining of glial fibrillary acidic protein (GFAP) was performed for the intrinsic cardiac ganglia (ICG), in posterior wall of left atrium^30^, to assess ICG neuroinflammation/dysregulation. The results provided a rich picture of persistent stress-induced cardiac malperformance upon chronic physical or psychological stress.

## Results

### Serum corticosterone concentration

The concentration of serum corticosterone exhibited a notable more than twofold increase in both the physically stressed group (2481.2 ± 156.8 ng/ml) and the psychologically stressed group (2430.8 ± 241.4 ng/ml) when juxtaposed with their respective control counterparts (1215.7 ± 120.4 ng/ml), clearly indicating successful stress induction (P < 0.01).

### Echocardiography

**Figure 2:**
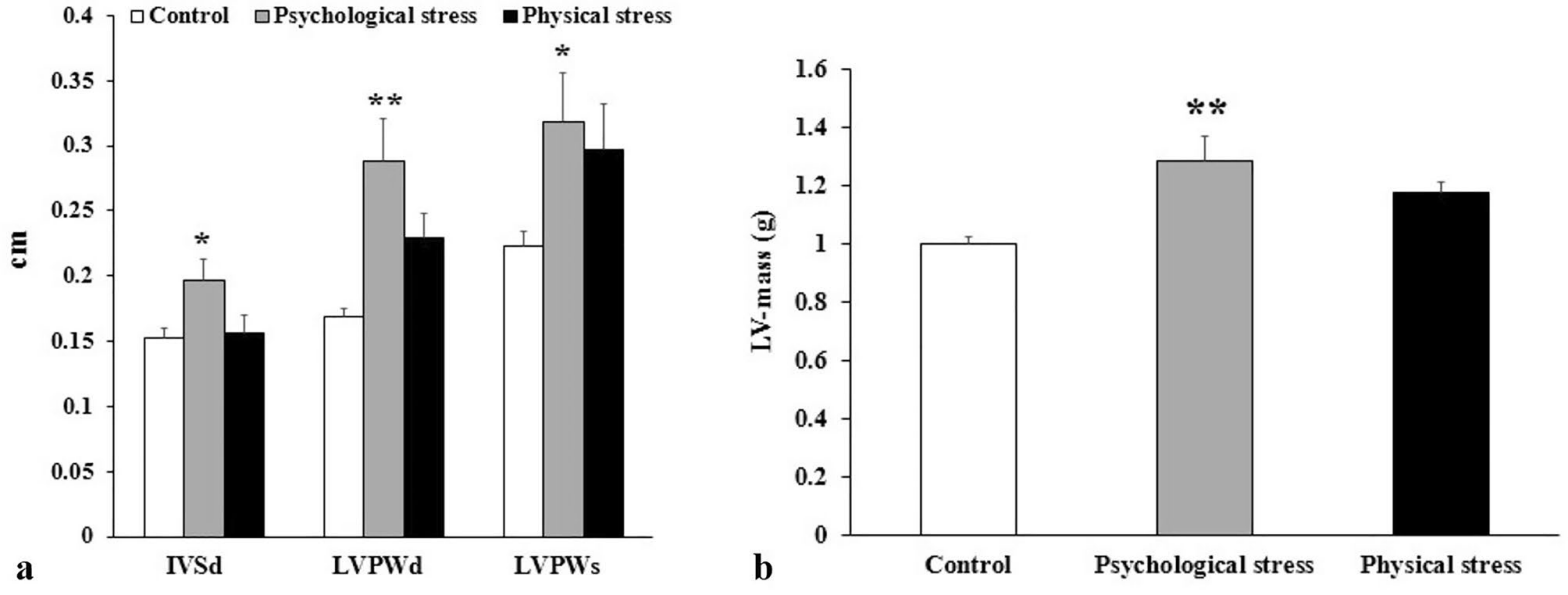
Changes in 4 out of 13 studied echocardiographic parameters. (**a**) Interventricular septum thicknesses in diastole (IVSd), left ventricular posterior wall thickness in diastole (LVPWd), and left ventricular posterior wall thickness in systolstole (LVPWs) exhibit statistically significant increase in the group of psychologically stressed compared to the control. (**b**) Left ventricular mass shows a significant increase in the group of psychologically stressed compared to the control. * and **represent p < 0.05 and p < 0.01, respectively. All data are expressed as mean ± SEM (N = 7 per group).

Statistical analysis of the echocardiography metrics, as summarized in Table 1, revealed noteworthy findings. Psychologically stressed rats displayed a significant augmentation in interventricular septum thicknesses in diastole (IVSd; P < 0.05), left ventricular posterior wall thickness in diastole (LVPWd; P < 0.01), left ventricular posterior wall thickness in systole (LVPWs; P < 0.05), and left ventricular mass (LVM; P < 0.01) in comparison to the control rats. However, physically stressed rats showed only minor, insignificant variations in their echocardiographic parameters (Fig. 2).

**Table 1:** Echocardiographic parameters.

Parameter means ± SEM	Control	Physical stress	Psychological stress	P-value
IVSd (cm)	0.1520 ± 0.0076	0.1565 ± 0.0133	0.1968 ± 0.0163*	0.019
LVDd (cm)	0.5335 ± 0.0160	0.5695 ± 0.0302	0.4875 ± 0.0308	0.180
LVPWd (cm)	0.1689 ± 0.0057	0.2297 ± 0.01834	0.2877 ± 0.0333**	0.002
IVSs (cm)	0.2559 ± 0.0090	0.2383 ± 0.0131	0.2802 ± 0.0161	0.186
LVDs (cm)	0.2875 ± 0.0255	0.3415 ± 0.0355	0.2608 ± 0.0330	0.528
LVPWs (cm)	0.2226 ± 0.0121	0.2967 ± 0.0348	0.3180 ± 0.0382*	0.015
LVEF (%)	81.750 ± 3.3530	74.833 ± 4.1103	83.167 ± 2.7010	0.760
FS (%)	46.625 ± 3.8680	40.000 ± 4.4572	48.333 ± 4.2480	0.773
DV (ml)	0.1621 ± 0.0162	0.2016 ± 0.0316	0.1285 ± 0.0226	0.237
SV (ml)	0.02899 ± 0.0068	0.04836 ± 0.0132	0.02275 ± 0.0061	0.524
Stroke volume (ml)	0.1331 ± 0.0118	0.1532 ± 0.0221	0.1057 ± 0.0167	0.193
CO (ml/min)	30.108 ± 1.9431	34.954 ± 4.6494	27.095 ± 4.2361	0.478
LV-mass (g)	1.000 ± 0.0241	1.175 ± 0.0378	1.283 ± 0.0836**	0.003

*IVSd* interventricular septum thicknesses in diastole, *LVDd* left ventricular diameter in diastole, *LVPWd* left ventricular posterior wall thickness in diastole, *IVSs* interventricular septum thicknesses in systole, *LVDs* left ventricular diameter in systole, *LVPWs* left ventricular posterior wall thickness in systole, *LVEF* left ventricular ejection fraction, *FS* fractional shortening, *DV* diastolic volume, *SV* systolic volume, *CO* cardiac output, *LV-mass* left ventricular mass. *^,^**Represented significant difference from control (P < 0.05 and P < 0.01, respectively).

### SPECT/CT

**Figure 3:**
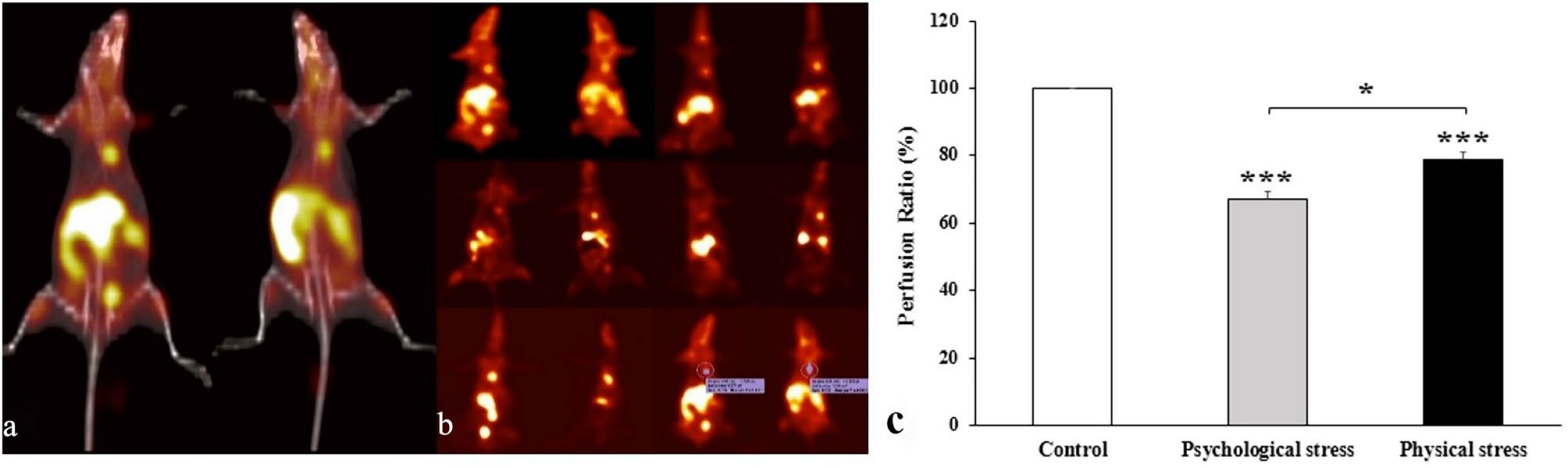
Myocardial perfusion with ^99^mTc-sestamibi. (**a**) SPECT/CT fused images of two rats 60 min after 1.5 mCi ^99^mTc-sestamibi injection show significant accumulation of radiotracer in kidneys, liver, heart and bladder, (**b**) SPECT single images of rats from different groups 45–90 min after radiotracer injection followed by 3D ROI analysis for uptake quantifications (last 2 images) demonstrate a substantial perfusion defect of 78.7%, indicating regional ischemia in psychologically stressed rats. This defect corresponds to perfusion ratios as low as 21.30%, (**c**) Myocardial perfusion ratio of 99mTc-sestamibi. ***p < 0.001 both stress groups vs. control, *p < 0.05 psychological stress vs. physical stress. Data are expressed as mean ± SEM (N = 3 per group).

Figure 3a presents fused SPECT/CT images of two rats (a psychologically stressed rat and a physically stressed rat) obtained using the previously described acquisition and reconstruction protocols. For enhanced clarity, SPECT images of rats from different groups 45–90 min after ^99^mTc-sestamibi injection are displayed in Fig. 3b. Moreover, the last two images showcase a sample 3D region of interest (ROI) analysis aimed at investigating heart-to-total body uptake ratios for each group. The 3D ROI analysis for uptake quantification unveiled substantial perfusion defect ratios in both stress groups (p < 0.001) when contrasted with the control group. Notably, the psychological stress group exhibited lower perfusion ratios compared to the physical stress group (21.30% versus 32.97%, p < 0.05) (Fig. 3c).

### Immunohistochemistry

**Figure 4:**
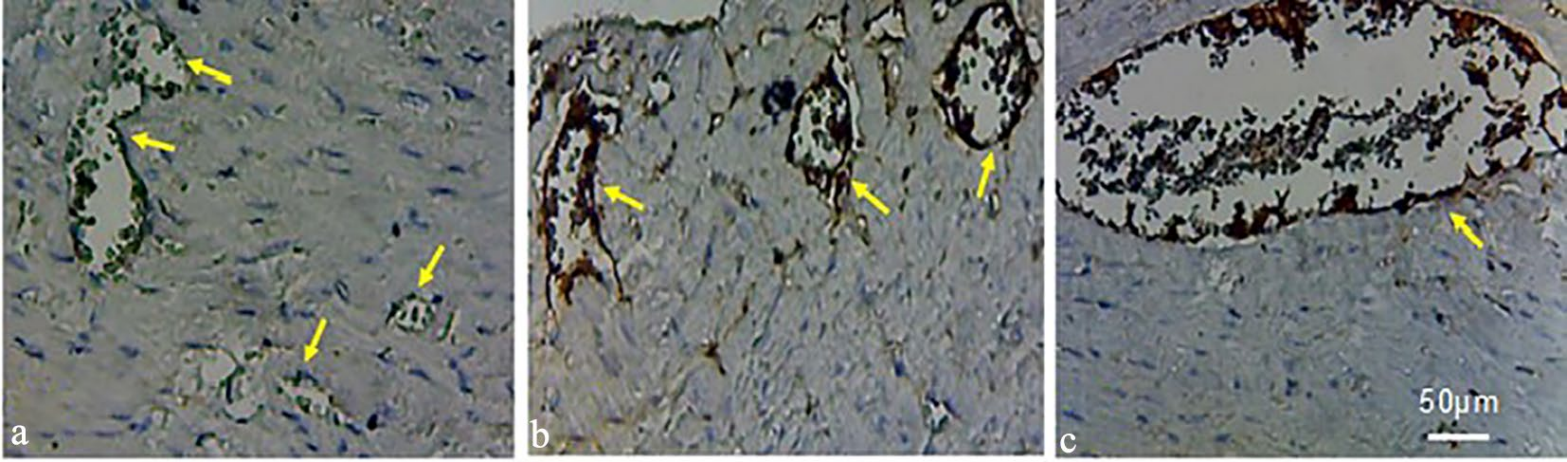
GFAP Immunohistochemical staining of posterior wall of left atrium. (**a**) Heart section of the control group demonstrated less GFAP. Both (**b**) physically and (**c**) psychologically stressed groups displayed more GFAP (neuroinflammatory responses). Psychological stress not only presented more expressions of GFAP, but also exhibited much larger cardiac ganglia. Arrows show the cardiac ganglia in each frame of tissue slices. Magnification × 20 (N = 7 per group).

In both stressed groups, the immunoreactivity of glial fibrillary acidic protein (GFAP) in the intrinsic cardiac ganglia (ICG) zone was prominently heightened in comparison to the control group. Specifically, the heart sections of psychologically stressed animals exhibited not only an increased density of GFAP-positive astrocytes but also noticeably larger cardiac ganglia (Fig. 4).

## Discussion

Stress stands as a significant detriment to well-being, with prior research documenting a robust connection between stress and cardiovascular disease (CVD), encompassing arrhythmias, heart failure, and myocardial ischemia^2–4^. The impact of stress on bodily functions is contingent upon the age^5^ and sex^35^ of the stressed organism. Furthermore, diverse forms of stress (e.g., acute/chronic, physical/psychological) are discerned by the brain through varying neural networks, resulting in distinct health complications^20^. While negative effects of psychological^17^ and physical stress^36^ on heart tissue have been recognized, an extensive comparative assessment of long-term effects from these distinct stressors on cardiac performance has remained elusive.

To address this gap, we exposed adolescent female rats to repeated physical or psychological stressors and analyzed their cardiac structural and functional parameters in adulthood. Although it is challenging to eliminate emotional effects of electric footshocks in the physically stressed group, the dominance of physical stress is evident.

The elevation in serum corticosterone levels on PND 39 solidified the stress response. In terms of the post-stress assessments in young adult rats, echocardiographic analysis indicated a significant increase in left ventricular thickness/mass in psychologically stressed rats, consistent with previous studies reporting left ventricular hypertrophy due to stress-induced elevated blood pressure^37^. Such hypertrophy is linked to a 2–fourfold increase in the risk of cardiovascular morbidity and mortality^38^. Hence, psychological stress, by inducing left ventricular hypertrophy, appears to pose a substantial risk to cardiovascular health. Notably, the significance of cardiac left ventricular hypertrophy was reaffirmed using two complementary techniques: cardiac CT scans and GFAP immunoreactivity assessment in the intrinsic cardiac ganglia (ICG) zone.

In the context of hybrid SPECT/CT imaging (high sensitivity) for myocardial ischemia evaluation, ^99^mTc-sestamibi, taken up by the myocardium proportional to blood flow in coronary arteries, displayed remarkable reductions in cardiac mitochondrial storage in psychologically stressed rats. This aligns with reports of myocardial ischemia resulting from psychosocial factors^39^. Furthermore, a reverse correlation between myocardial ^99^mTc-sestamibi storage and atrial natriuretic factor expression, a heart failure marker^40^, indicates a higher CVD risk in psychological stress compared to physical stress.

The immunohistochemistry staining results indicated increased GFAP expression in the ICG zone of both stressed groups, reflecting astrocyte reactivity and ICG neuroinflammation/dysregulation^41–43^. Regardless of other histopathological and immunohistochemical changes of the heart tissue, our main focus of GFAP staining of posterior wall of left atrium, as the main location of cardiac ganglia, was to investigate the issue on how activation of different neural networks triggered by physical or psychological stress has affected the cardiac neural ganglia. Notably, cardiac tissue slices of psychologically stressed rats exhibited significantly higher GFAP-positive astrocytes and larger cardiac ganglia. This observation aligns with studies linking myocardial ischemia to increased inflammatory responses^44,45^, which can affect ICG through accumulated myocardial tissue chemicals^46^. Interestingly, the heart sections of psychologically stressed rats displayed notably larger cardiac ganglia^47^. In line with a study on age-related ICG architecture^48^, this could suggest that ICG development is hindered in psychologically stressed rats, a novel finding requiring further exploration.

Considering the role of the intrinsic cardiac nervous system (ICNS) in regulating cardiac conduction systems^49^, the greater ICG dysregulation in psychologically stressed rats could imply a higher susceptibility to arrhythmias compared to physically stressed rats. This notion gains support from our previous study's results, indicating more irregular rhythm and heart rate variability in psychologically stressed rats compared to physically stressed ones^50^.

The results collectively suggest that psychological stress induces left ventricular mass and thickness increase, cardiac hypertrophy, myocardial ischemia, and ICG neuroinflammation/dysregulation, while physical stress shows insignificant associations with these outcomes. Remarkably, the larger ICG size in psychologically stressed rats unveils a new dimension requiring further investigation.

It is noteworthy to mention if the stress-induced pathological changes are detectable on PND 80–83, these pathologic modifications could be considered almost persistent; since the time interval between stress induction period and post-stress tests is nearly equivalent to 4.5 years in humans^51^.

It is pertinent to acknowledge the study's limitations. SPECT/CT was conducted with a clinical scanner (Siemens, Germany). Although our findings were corroborated by previous results, larger SPECT/CT studies are recommended. Additionally, the emotional impact of electric footshock in the physically stressed group cannot be entirely eliminated. However, the observation of lower physiological changes in the physically stressed group than in the psychological stress group is noteworthy. Future research should address these limitations.

## Conclusion

This study's statistically significant changes in cardiac performance parameters in psychologically stressed rats underscore the link between adolescent psychological stress and future cardiovascular disease incidence. In contrast, this relationship appears negligible with adolescent physical stress. The findings emphasize the importance of stress management^52^ and psychological healthcare^53^ during adolescence as a preventive strategy to reduce future CVD prevalence. Moreover, improved quality of life among CVD patients could result from this approach. These conclusions align with evidence supporting the substantial contribution of positive psychological factors, such as emotional vitality^54^, optimism^55,56^, and positive affect^57^, in reducing cardiovascular risk and enhancing composite cardiovascular health scores. Given the pivotal role of the intrinsic cardiac nervous system (ICNS) in cardiac function, future research should explore histological and molecular approaches to decipher the mechanisms underlying long-term psychological stress effects on cardiac neural pathways.

## Data Availability

The datasets used and/or analyzed during the current study available from the corresponding author on reasonable request.
